# Supplementary material for: An examination of the prevalence of metabolic syndrome in older adults in Ireland: Findings from The Irish Longitudinal Study on Ageing (TILDA)
Source: PLoS One. 2022 Sep 14;17(9):e0273948. doi: 10.1371/journal.pone.0273948 (PMC9473442; doi:10.1371/journal.pone.0273948)
Supplement: S1 Table — Notes: Data presented as weighted proportions with percentages with 95% confidence intervals in brackets. TILDA, The Irish Longitudinal Study on Ageing; SHARE, Survey of Health, Ageing and Retirement in Europe; Body mass index (BMI) measured by self-report in SHARE; Overweight = BMI ≥25kg/m2 & <30kg/m2; Obese = BMI≥30kg/m2; Overweight/obese = ≥25kg/m2. a BMI calculated using measured height and weight; b BMI calculated from self-reported height and weight. (DOCX) [file pone.0273948.s001.docx]

**S1 Table:** **Comparison of prevalence of overweight/obese as measured by body mass index**

|  | **TILDA^a^ n=5340** | **SHARE^b^ n=35,428** |
| --- | --- | --- |
| **Overweight** | 42.5% (41.0, 44.0) | 41.3% (40.5, 42.1) |
| **Obese** | 34.5% (33.1, 35.9) | 19.2% (18.3, 20.2) |
| **Overweight/obese** | 77.0% (75.7, 78.3) | 60.5% (59.9, 61.2) |
| **Age** | 63.7 (63.3, 64.1) | 66.6 (66.5, 66.7) |

Notes: Data presented as weighted proportions with percentages with 95% confidence intervals in brackets. TILDA, The Irish Longitudinal Study on Ageing; SHARE, Survey of Health, Ageing and Retirement in Europe; Body mass index (BMI) measured by self-report in SHARE; Overweight = BMI ≥25kg/m^2^ & <30kg/m^2^; Obese = BMI≥30kg/m^2^; Overweight/obese= ≥25kg/m^2
a^ BMI calculated using measured height and weight; ^b^ BMI calculated from self-reported height and weight
